# Postoperative functional training program for vascularised Iliac flap donor site in jaw defect reconstruction based on the Delphi method

**DOI:** 10.1038/s41598-025-13774-x

**Published:** 2025-08-02

**Authors:** Li Li, Qian He, Na Zhou, Zhaoxia Zhang, Xiaoming Lv, Jie Zhang

**Affiliations:** Department of Oral and Maxillofacial Surgery, Peking University School and Hospital of Stomatology & National Center of Stomatology & National Clinical Research Center for Oral Diseases & National Engineering Research Center of Oral Biomaterials and Digital Medical Devices, No. 22 Zhongguancun South Avenue, Haidian District, Beijing, 100081 China

**Keywords:** Vascularised iliac flap, Delphi method, Functional training, Nursing, Diseases, Health care, Health occupations, Oncology, Risk factors

## Abstract

Vascularised iliac flaps (VIFs) are widely used for the reconstruction of jawbone defects; however, postoperative donor-site complications, such as gait disturbances, with an incidence of 13.9–50%, significantly impede patient recovery. Despite this, evidence-based rehabilitation protocols specific to VIFs remain lacking. Existing rehabilitation guidelines for hip surgeries are unsuitable owing to differences in surgical mechanisms. This study employed the Delphi method, engaging 20 multidisciplinary experts (oral and maxillofacial surgery: 5; orthopaedics: 7; rehabilitation: 6; nursing: 2). Through three rounds of anonymous consultations, and by integrating literature evidence with postoperative mobility assessments, we developed a phased, individualised progressive functional training (PFT) protocol featuring dynamic evaluation, coordinated activation of abdominal and hip muscle groups, and safe exercise strategies during head and neck immobilisation, while overcoming conventional hip rehabilitation limitations (e.g., restrictions on flexion < 90°, and bans on squatting or cross-legged sitting). PFT is structured into six progressive phases, with exercise intensity tailored to assessment outcomes. A single-centre randomised controlled trial (n = 62) demonstrated that PFT significantly accelerated lower limb functional recovery, improved hip mobility and balance, reduced donor-site pain, and enhanced quality of life (University of Washington Quality of Life questionnaire: F (1,60) = 17.262, *P* < 0.001), without increasing the risk of flap vascular compromise or iliac hematoma. The limitations of the study include the single-centre design and lack of cross-cultural validation. Future multicentre studies are required to enhance adaptability. This study establishes a foundational yet effective framework for post-VIF rehabilitation, guiding clinical practice and research advancements.

## Introduction

Vascularised iliac flaps (VIFs) are widely utilised in the reconstruction of jawbone defects owing to their abundant bone volume, anatomical curvature, compatibility with the mandible, and ability to carry soft tissue components^1–4^. However, the surgical procedure compromises fascial integrity and involves detachment of the external oblique, internal oblique, transversus abdominis, and iliopsoas muscle attachments. Postoperative donor-site complications include pain, paraesthesia, thigh numbness, gait disturbances, and inguinal hernia. Among these, gait disturbance represents the most prevalent complication, with an incidence ranging from 13.9% to 50%. Some patients experience prolonged walking difficulties, restricted lumbar mobility, and delayed hip functional recovery that may persist for 6 months to 5 years^5–10^.

Despite advancements in surgical techniques to reduce complication rates^3,10^, there remains a critical gap in evidence-based rehabilitation protocols specifically addressing donor-site complications after VIF procedures^5,6^. Clinical observations indicate that oncology patients frequently exhibit reduced physical activity owing to fear of pain, while inadequate post-discharge guidance exacerbates anxiety, increasing the risk of long-term sequelae^11,12^. Existing rehabilitation guidelines for hip fractures or joint arthroplasty are not suitable for VIF reconstruction, as these procedures typically involve only the fracture site or joint cavity, resulting in minimal tissue damage, and do not require extensive resection of abdominal wall musculature^13,14^. In contrast, VIF reconstruction involves harvesting larger bone segments (4.0–12.5 cm in length, 2.0–3.0 cm in height, and 1.0–1.5 cm in thickness) and abdominal wall muscles (20–56 cm^2^), leading to significantly increased hip wound tension^8,15^. As such, functional training must address three critical considerations: (i) postoperative mobility is significantly influenced by the dimensions (length and volume) of the harvested bone^8,16^, necessitating individualised training protocols; (ii) targeted training is required to strengthen the abdominal and hip muscle groups; and (iii) strict immobilisation of the head and neck is required to protect the vascular anastomosis between the deep circumflex iliac vessels and cervical vasculature^17^.

This study aimed to develop a specific progressive functional training (PFT) protocol for VIFs. In contrast to traditional hip surgery rehabilitation protocols, this programme introduces three key innovations: (i) progressive and individualised training based on dynamic assessment; (ii) a strategy for the coordinated activation of the abdominal wall and hip muscle groups, with targeted strengthening of core stabilisers such as the transversus abdominis and iliopsoas muscles; and (iii) a safe exercise regimen during the head and neck immobilisation period to facilitate early rehabilitation while protecting the vascular anastomosis. Unlike traditional hip rehabilitation protocols that restrict hip flexion to ≤ 90°, ^18^ PFT allows unrestricted flexion angles during hip mobility training, enabling patients to perform functional movements such as squatting and sitting cross-legged. This approach is designed to accelerate the recovery of hip joint and lower limb functions and improve patients’ quality of life.

## Methods

### Study design

This study employed the Delphi method to develop the VIF rehabilitation protocol, guided by two principal considerations. First, the Delphi technique facilitates anonymous, multi-round expert consultations, effectively mitigating authority bias and groupthink. Unlike nominal group techniques, where dominant experts may disproportionately influence discussions, or brainstorming methods that prioritise creative ideation over rigorous consensus-building, the Delphi approach demonstrates superior applicability for complex clinical decision-making^19,20^. Second, its iterative feedback mechanism allows experts to maintain independence in expressing opinions while progressively integrating multidisciplinary perspectives from surgery, nursing, and rehabilitation, which is a critical requirement for developing a cross-disciplinary protocol^21,22^. The study was conducted in accordance with the recommendations outlined in the ‘Guidance on Conducting and Reporting Delphi Studies (CREDES)^23^.

### Expert panel

In this study, 20 experts were selected to participate in the Delphi consultation. This sample size was chosen to balance disciplinary diversity with the efficiency of opinion synthesis, thereby minimising the difficulties in achieving consensus that often accompany larger panels^24^. The expert composition (oral and maxillofacial surgery, orthopaedics, rehabilitation, and nursing in a 5:7:6:2 ratio) directly addressed the multidisciplinary needs of VIF rehabilitation. Oral and maxillofacial surgeons oversaw surgical protocols and complication management, orthopaedic specialists evaluated donor-site biomechanical stability and preventive care^25,26^, rehabilitation experts developed functional recovery protocols, and nursing professionals ensured clinical implementation and integration of patient education.

Expert selection was based on strict criteria to ensure representativeness and credibility. Regional representation was ensured by recruiting experts from 11 tertiary hospitals covering diverse geographic regions: North China (2), East China (2), South China (2), Central China (2), Southwest China (1), Northwest China (1), and Northeast China (1) to eliminate geographic bias. Furthermore, professional qualifications required direct involvement in ≥ 30 VIF postoperative rehabilitation cases (including surgical, rehabilitative, and nursing roles) within the past 5 years, ≥ 10 years of clinical experience, a bachelor’s degree or higher, intermediate or senior professional titles, and a commitment to completing all three Delphi consultation rounds.

### Initial version of the functional training programme

The initial version of the PFT protocol was collaboratively formulated by a seven-member team, drawing upon literature evidence and postoperative patient mobility assessments. Four nurses searched the relevant literature published from 1990 to 2022 across several databases, including CNKI, PubMed, SinoMed, Web of Science, EBSCO, the Cochrane Library, and Nature. The primary search strategy included the terms: “vascularised iliac flap/deep circumflex iliac artery free flap/ilium transplantation/iliac bone graft/mandibular reconstruction” AND “donor site morbidity/complication” AND “exercise rehabilitation/physical activity/function training/nursing”. There was only one study on functional exercises following alveolar bone grafting with the ilium, which focused on hip joint abduction, flexion, and gait exercises^27^. To broaden the scope, the databases were then searched using the terms “hip/coxa” AND “exercise rehabilitation/physical activity/function training/nursing/guideline/consensus/recommendation”. The initial search yielded 1,276 articles, including 612 duplicates. After abstract and full text screening, eight articles were included for data extraction. The key points of the functional exercises are summarised in Table 1. The need for a VIF-specific rehabilitation protocol was based on two key differences: while hip surgeries often involve hip joint injury or prosthesis implantation, VIF surgery preserves hip joint integrity; second, hip surgeries do not require extensive harvest of abdominal muscle and iliac bone, whereas VIF reconstruction involves harvesting relatively large bone segments (4.0–12.5 cm in length, 2.0–3.0 cm in height, and 1.0–1.5 cm in thickness) and abdominal muscles (20–56 cm^2^), leading to considerably increased hip wound tension. Accordingly, the PFT protocol was designed to address these unique demands by focusing on: (i) unrestricted hip flexion, allowing functional postures such as squatting and sitting cross-legged; (ii) coordinated activation of abdominal and hip muscle groups, with targeted training of core stabilisers (e.g., transversus abdominis, iliopsoas); and (iii) safe exercise protocols during head and neck immobilisation to balance vascular anastomosis protection with early-stage rehabilitation. The initial version of the PFT protocol was developed based on both the available evidence and clinical observations of early postoperative mobility in patients undergoing VIF reconstruction^16^.

**Table 1:** Summary of rehabilitation recommendations after hip surgery.

	Content
Timing	Exercise in or out of bed within 24 h after operation^27,37,38^
Frequency	At least once a day^38,39^
Method	All limbs, including the surgical limb, need to be exercised Exercising, walking, and daily life activities should be emphasised Exercises include hip joint activities, hip muscle strength training, gait training, and weight-bearing or progressive resistance training^31,37–42^
Evaluation	Harris Hip Score and independent bed chair transfer for assessing mobility^38–40^
Continuation	Functional exercises continue from the hospital to the family or community^38–40^

### Questionnaire

The expert consultation questionnaire comprised four sections: (i) an introduction outlining the research background, objectives, completion instructions, and feedback deadline; (ii) expert demographic information (age, gender, education, professional title, years of experience, and specialty); (iii) an authority assessment section evaluating each expert’s familiarity with the subject matter and their judgement criteria; and (iv) the core content, which included assessment indicators rated for importance and feasibility using a 5-point Likert scale (1 = very low, 5 = very high). Experts were also granted the authority to propose professional and practical modification suggestions.

### Expert consultation

**Fig. 1:**
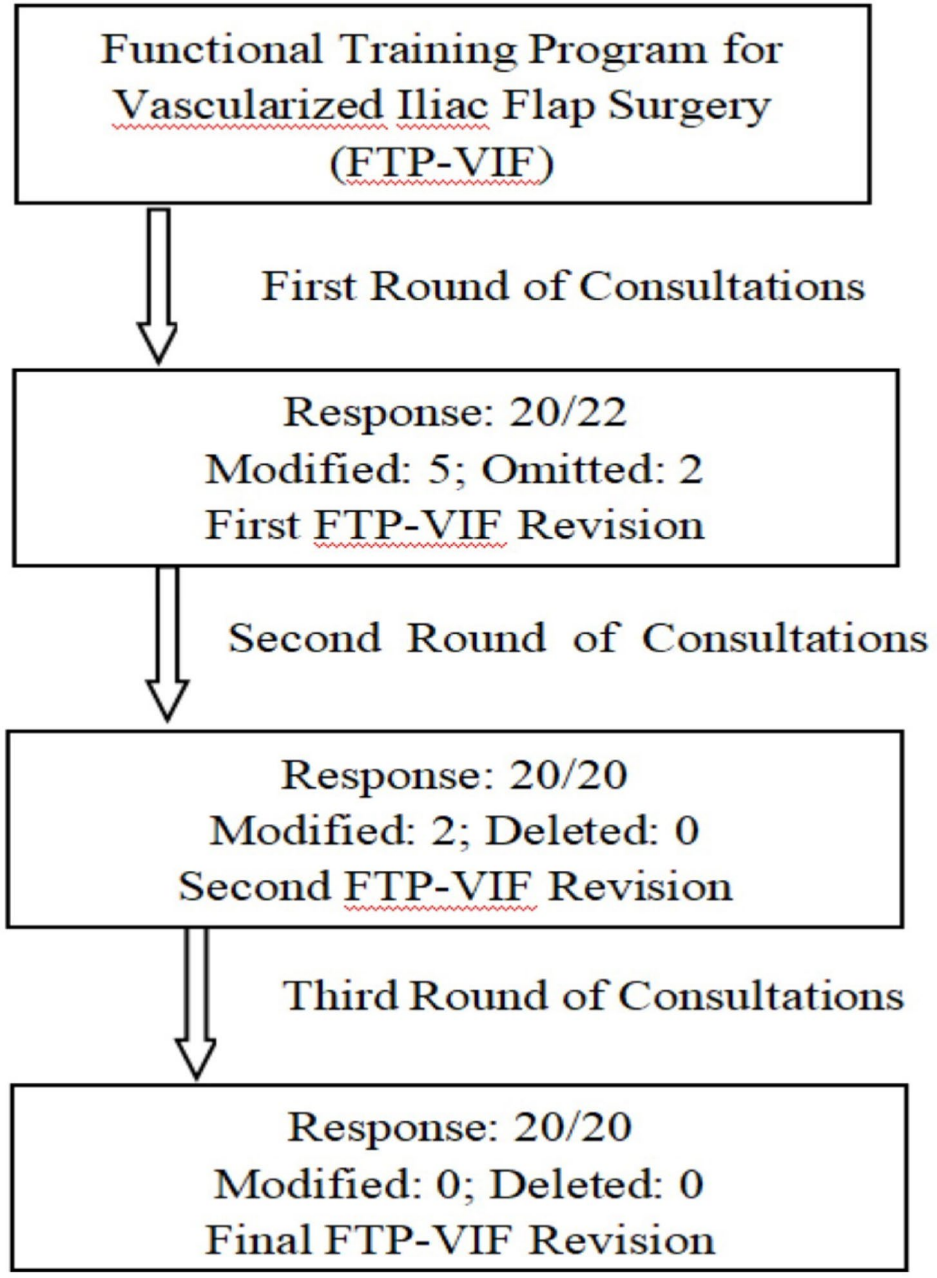
Flowchart of Delphi method.

This study employed a modified Delphi method with three rounds of expert consultation. Electronic questionnaires were distributed to 22 experts through WeChat or email from October to December 2022. The experts were asked to rate the importance and feasibility of each evaluation dimension and item using a 5-point Likert scale and to provide written suggestions for improvement. The research team conducted an integrated analysis of both quantitative ratings and qualitative feedback. Items were retained if they met predefined criteria: a mean score > 4.00 and a coefficient of variation < 0.25^28^. Based on this analysis, a revised questionnaire was developed for the next round. In each round, the experts were provided with feedback on the consensus results and the basis for any revisions from the previous round. This iterative optimisation process facilitated the achievement of a stable consensus, leading to the finalisation of the PFT protocol. The detailed revision process is illustrated in the workflow diagram (Fig. 1).

### Statistical analysis

Quantitative and hierarchical variables were analysed using IBM SPSS Statistics for Windows, version 24.0 (IBM Corp., Armonk, NY, USA). Descriptive statistics were used to summarise the basic demographic information of the experts. Expert assessment metrics included the questionnaire recovery rate, the authority coefficient of the experts or composite reliability (Cr), coefficient of variation (CV), and Kendall’s coefficient of coordination (W). The importance and feasibility of each rehabilitation item were described as means ± standard deviations.

### Ethics statement

The study was conducted in accordance with the Declaration of Helsinki and was approved by the Ethics Committee of Peking University School of Stomatology (Approval No. PKUSSIRB-202278107). Written informed consent was obtained from all participants before enrolment.

## Results

### Expert positivity and authority

In the first round of the Delphi survey, 2 experts did not respond; however, all 20 experts participated in the subsequent two rounds. Detailed information regarding the consulted experts is provided in Table 2. The effective response rates across the three rounds of questionnaires were 90.91%, 100%, and 100%, respectively. The adoption rates of expert suggestions were 65% in the first round, 55% in the second, and 27.78% in the third, indicating strong engagement and acceptance. The experts demonstrated high authority, with a Cr score of 0.86 and CVs ranging from 0.10 to 0.20. The Kendall’s W coefficients for the three rounds were 0.09, 0.12, and 0.19, respectively. Although these W-values appear relatively low (potentially owing to the large expert cohort of > 15 participants), all *P*-values were < 0.001, confirming statistically significant consensus. Notably, the W-values showed a progressive improvement across rounds, reflecting improving agreement among experts^28^.

**Table 2:** Basic information of Delphi correspondence experts (n = 20).

Item	Classification	Constituent ratio
Years of experience	10–14	0.75
	≥ 15	0.25
Education	Master’s degree or below	0.40
	Doctoral degree	0.60
Professional title	Intermediate	0.35
	Senior	0.55
Work department	Oral and maxillofacial surgery	0.25
	Rehabilitation	0.35
	Orthopaedics	0.30
	Nursing	0.10

### Delphi rounds and framework modifications

*Round 1*.

Recommendations proposed by 13 experts (65.00%) were incorporated into the protocol revision following a comprehensive evaluation by the research team. The training protocol was modified as follows:

① Phased Framework & Assessment Mechanism: The overall training period was divided into six stages (A–F). Functional capacity assessments were conducted at the end of each stage, with progression to the next stage based on assessment results. Exercise volume and intensity increased gradually with each stage; ② Training Content Enhancement: living ability training (sit-ups, standing training, toilet training, sitting training) and balance training were added to improve functional independence; ③ Frequency Adjustment: Restrictions on training frequency in A–C stages were removed, with patient tolerance prioritised as the primary criterion. After achieving independent ambulation, the total daily training time was increased to ≥ 30 min; and ④ Deleted Content: Knee and hip joint mobility exercises during the bed rest stage.

*Round 2*.

Of the 20 experts, 11 (55.00%) provided suggestions for improvement in the final round. The primary change adopted was the advancement of two training components (bridge exercises and hip muscle strengthening) from the independent walking stage to the transition stage. The experts were provided with justifications for any suggestions that were not adopted. No new items were added or omitted during this round. All items met the predefined retention criteria for importance and feasibility, supporting the validity and reliability of the study.

*Round 3*.

The experts generally had consistent opinions, with no major differences in their responses. All retained items exhibited CV of 0.15–0.20 for importance ratings and 0.16–0.20 for feasibility ratings. Therefore, the Delphi consultation process was concluded, resulting in the finalisation of the definitive version of the PFT protocol (Table 3).

**Table 3:** Progressive functional training for vascularised iliac flaps (n = 20).

Training phase and content	Importance		Feasibility	
	x ± s	CV	x ± s	CV
A Bed rest stage (1–3 days postoperatively)	4.72 ± 0.45	0.15	4.89 ± 0.31	0.18
A1 Position: supine or semi-sitting	4.56 ± 0.68	0.15	4.78 ± 0.63	0.19
A2 Basic training: ankle pump exercise, quadriceps isometric contraction, and abdominal breathing	4.72 ± 0.56	0.16	5.00 ± 0.00	0.20
A3 Living ability training: sit-ups (paying attention to head braking)	4.78 ± 0.42	0.16	4.89 ± 0.31	0.18
*Evaluation: The patient keeps sitting for 1 min, and then lifts the affected limb off the bed surface*	5.00 ± 0.00	0.20	4.78 ± 0.63	0.19
B Assisted standing and walking stage (4–7 days postoperatively)	5.00 ± 0.00	0.20	4.78 ± 0.42	0.16
B1 Hip activity training in supine position (ROM without restrictions)	4.67 ± 0.58	0.15	5.00 ± 0.00	0.20
B2 Basic training: Continued A2 protocol	4.78 ± 0.42	0.16	5.00 ± 0.00	0.20
B3 Living ability training: standing/toilet/sitting training (pay attention to head braking)	5.00 ± 0.00	0.16	5.00 ± 0.00	0.20
*Evaluation: Independent ambulation/standing* > *1 min; VAS* ≤ *4; no active bleeding at iliac crest incision*	5.00 ± 0.00	0.20	4.78 ± 0.63	0.20
C Transition stage (8–14 days postoperatively)	5.00 ± 0.00	0.20	5.00 ± 0	0.19
C1 Hip activity training in standing/sitting positions (ROM without restrictions)	4.78 ± 0.42	0.16	5.00 ± 0.00	0.20
C2 Abdominal core muscles and pelvic stability: Supine hip lift/bridge (abdominal breathing)	4.78 ± 0.42	0.16	5.00 ± 0.00	0.20
C3 Living ability training: walk training (body weight shifts from the healthy to the affected limb)	5.00 ± 0.00	0.20	5.00 ± 0.00	0.20
*Evaluation: Full weight-bearing ambulation; no bleeding/swelling/pain at iliac crest incision*	5.00 ± 0.00	0.20	5.00 ± 0.00	0.20
D1 Hip muscle strength training: Weight-bearing or elastic band training (ROM without restrictions)	4.83 ± 0.37	0.20	5.00 ± 0.00	0.20
D2 Abdominal core muscles and pelvic stability: Continued C2 protocol	5.00 ± 0.00	0.17	5.00 ± 0.00	0.20
D3 Balance training: Turning/circle/straight ambulation; high-step negotiation; single-leg stance (healthy → affected)	5.00 ± 0.00	0.20	5.00 ± 0.00	0.20
D1 Hip muscle strength training: Weight-bearing or elastic band training (ROM without restrictions)	5.00 ± 0.00	0.20	4.89 ± 0.31	0.20
*Evaluation* *: * *Achieved bidirectional turns within 4 s, 8-step alternating ascent in 20 s, and 2-min single-leg stance on healthy limb*	5.00 ± 0.00	0.20	5.00 ± 0.00	0.20
E Independent living stage (1–2 months postoperatively)	4.61 ± 0.68	0.20	5.00 ± 0.00	0.18
E1 Hip muscle strength training: Continued D1 protocol, increased weight-bearing per tolerance (full ROM)	5.00 ± 0.00	0.16	5.00 ± 0.00	0.20
E2Abdominal core muscles and pelvic stability: Bridge + high horse stance with abdominal breathing	4.94 ± 0.23	0.20	5.00 ± 0.00	0.20
E3 Balance training: stand on the affected limb and try to step forward, backward, and sideways	4.94 ± 0.23	0.19	5.00 ± 0.00	0.20
E4 Living ability training: going up and down stairs, sweeping the floor, etc	5.00 ± 0.00	0.19	4.89 ± 0.31	0.20
*Evaluation: stand on the affected limb for at least 2 min, pick up objects from the ground, or TUGT* < *20 s*	5.00 ± 0.00	0.20	5.00 ± 0.00	0.20
F Return to society (> 2 months postoperatively)	4.83 ± 0.37	0.20	5.00 ± 0.00	0.18
F1 Hip muscle strength training: Further weight-bearing individualized to capacity (full ROM)	5.00 ± 0.00	0.17	5.00 ± 0.00	0.20
F2 Abdominal core muscles and pelvic stability training: Transitioned high horse stance^2^ → squats	4.94 ± 0.23	0.20	4.89 ± 0.31	0.20
F3 Balance training: Jogging/brisk walking with progressive distance	4.94 ± 0.23	0.19	5.00 ± 0.00	0.20
F4 Living ability training: resume daily work and life	5.00 ± 0.00	0.19	5.00 ± 0.00	0.18
*Evaluation: Functional training may be discontinued at 3 months if the patient meets all criteria: Harris score* > *95; TUGT* < *10 s or at preoperative level; no claudication; no chronic iliac wound pain. Otherwise, continue F-phase training*	4.72 ± 0.45	0.20	5.00 ± 0.00	0.20

ROM: range of motion; VAS: Visual analogue scale (pain); TUGT: Timed up and go test.

Training frequency A-B stage: Unlimited frequency, subject to patient tolerance.

C-F stage: Each item 10–15 times, or at least 30 min a day, depending on patient tolerance^1^.

^1^Patient tolerance: VAS < 4 points and a perceived fatigue level (rating of perceived exertion) of < 17 points.

^2^High horse stances: Shoulder-width squat during exhalation; knees posterior to toes, thighs subparallel; optional chest-crossed hands.

## Discussion

This study developed a PFT protocol for patients undergoing VIF reconstruction by integrating early postoperative mobility assessments with expert consensus derived through the Delphi method. A single-centre randomised controlled trial (RCT) involving 62 participants demonstrated the safety and efficacy of the protocol. The following section interprets the findings in relation to existing literature, discusses methodological limitations, and proposes future research directions^29^.

### Innovations and advantages of the PFT protocol

Postoperative rehabilitation challenges following VIF reconstruction primarily stem from donor-site complications and the high individual variability in functional recovery—particularly as bone graft volume directly influences postoperative mobility and pain levels^8,16^. Conventional rehabilitation protocols, which often rely on fixed-timeline models^30^, struggle to adapt to diverse patient recovery trajectories. In contrast, the PFT protocol employs an individualised, progressive, and dynamically assessed approach to systematically address three key domains:

(i) *Optimisation of hip joint mobility*: The PFT protocol employs a “passive–active–resistive” progression strategy, transitioning from supine range-of-motion exercises to weight-bearing resistance training after wound healing. This staged approach improves gait velocity, promotes muscle hypertrophy, and enhances overall hip joint function^31^.
(ii) *Enhancement of core stability*: Given that the integrity of abdominal wall musculature is often compromised during VIF harvesting—thereby increasing the risk of abdominal hernia^6^—and that some patients experience tightness in the iliolumbar region^5^, the protocol incorporates abdominal and iliolumbar musculature training (e.g., abdominal breathing, bridge exercises). These interventions follow progressive resistance training principles of muscle remodelling, reducing the incidence of hernia and improving postural control by increasing muscle elasticity and thickness^31–35^.
(iii) *Functional integration*: Balance training was incorporated to strengthen lower limb function and prevent falls^35,36^. In addition, task-oriented exercises (e.g., stair climbing, floor sweeping) were introduced to accelerate the recovery of activities of daily living. Throughout the program, strict axial stabilisation of the head and neck was maintained to protect the vascular anastomosis and mitigate the risk of vascular compromise.

### Efficacy and safety of the PFT protocol

At our centre, 62 patients were randomly assigned in a 1:1 ratio using a computer-generated allocation list to either the control group or the intervention group. The control group received routine hospital care, including sit-to-stand transitions, initiation of ambulation, and walking aid-assisted gait training, but no structured post-discharge rehabilitation guidance. In contrast, the intervention group underwent protocol-based rehabilitation following the PFT protocol. The RCT confirmed that the PFT program can accelerate the recovery of hip joint mobility and balance capacity, while reducing donor site pain. Notably, the intervention led to significant improvements in University of Washington Quality of Life (UW-QOL, version 4.0) scores compared to the control group (F(1,60) = 17.262, *P* < 0.001, η^2^ = 0.223). Furthermore, a significant interaction was observed between time and training modality (F(4,57) = 4.084, *P* = 0.006, η^2^ = 0.223), indicating that the benefits of PFT on quality of life become more pronounced over time, eventually surpassing those seen in the control group. Importantly, no significant differences were observed between the groups in terms of the incidence of flap vascular compromise, iliac hematoma or seroma, iliac fracture, or ventral hernia, confirming the safety of the PFT protocol^29^.

### Limitations and future research directions

First, all experts involved in this study were affiliated with domestic medical college hospitals, and the lack of international or multicentre experience may limit the generalisability of the PFT protocol to different medical environments and ethnic populations. Future studies should include international experts to validate the protocol’s cross-cultural adaptability. Second, although the single-centre RCT ensures high internal validity, its external validity may be constrained by potential confounding factors such as regional differences in patient physical constitution (e.g., northern vs. southern populations) and variability in healthcare resource accessibility, including equipment availability.

Future research should focus on large-scale, multicentre RCTs, particularly those including European and American centres, to enhance the generalisability and cross-cultural applicability of the PFT protocol. Regarding protocol optimisation, tailored adjustments, such as simplified exercise regimes for elderly patients, are recommended to improve feasibility and adherence across diverse patient populations. From a technological perspective, the integration of telerehabilitation tools, including remote video guidance and wearable devices capable of real-time monitoring of joint range of motion and muscle activity, holds promise for standardising training delivery while reducing the burden of hospital visits.

## Conclusion

The PFT developed through the Delphi method offers a preliminary yet functionally effective approach to postoperative rehabilitation following VIF transplantation. It establishes a reference framework to guide subsequent clinical rehabilitation practices and research endeavours in this specialised field.

## Data Availability

The datasets generated and/or analyzed during the current study are available from the corresponding author upon reasonable request.

## Abbreviations

VIFs: Vascularised iliac flaps
PFT: Progressive functional training
CV: Coefficient of variation
RCT: Randomised controlled trial
UW-QOL: University of Washington Quality of Life questionnaire (version 4.0)
VAS: Visual analogue scale (pain)
ROM: Range of motion
TUGT: Timed up and go test
